# Metabolic Syndrome Is Associated with Low Diet Quality Assessed by the Healthy Eating Index-2015 (HEI-2015) and Low Concentrations of High-Density Lipoprotein Cholesterol

**DOI:** 10.3390/biomedicines10102487

**Published:** 2022-10-05

**Authors:** Klaudia Konikowska, Wojciech Bombała, Andrzej Szuba, Dorota Różańska, Bożena Regulska-Ilow

**Affiliations:** 1 Department of Dietetics, Wroclaw Medical University, Borowska Street 211, 50-556 Wroclaw, Poland; 2 Statistical Analysis Center, Wroclaw Medical University, Marcinkowski Street 2-6, 50-368 Wroclaw, Poland; 3 Department of Angiology, Hypertension and Diabetology, Wroclaw Medical University, Borowska Street 213, 50-556 Wroclaw, Poland

**Keywords:** metabolic syndrome, diet, diet quality, nutrition, HEI-2015

## Abstract

Presenting diet quality of patients with metabolic syndrome (MetS), using a holistic approach is more useful than investigating dietary individual components, but there is still a small amount of research in this area. The aim of this study assessed the diet quality, as measured by the HEI-2015, of MetS patients compared to healthy individuals. The study and control group consisted of 215 patients with MetS and 320 people without MetS, respectively. A nutritional analysis using a semi-quantitative food frequency questionnaire was used to evaluate the nutritional habits in the study and control group. Total HEI-2015 scores were significantly lower in MetS subjects than in those in the control group (65.04 ± 9.71 vs. 66.75 ± 8.88) and the quality of women’s diets was better than the quality of men’s diet (66.83 ± 8.99 vs. 64.75 ± 9.57). We also observed that low HDL-c concentration increased the risk of MetS in the general population the most. Across the population, there was a weak positive correlation between HDL-c concentrations and total HEI-2015 scores and a weak negative correlation between mean waist circumference values and total HEI-2015 scores. HDL-c concentrations may be a key factor in the prevention of MetS and appropriate therapeutic management to increase HDL-c levels may be of key importance in patients diagnosed with MetS.

## 1. Introduction

The implementation of nutritional recommendations plays an essential role in the prevention of diet-related diseases, as well as in overall lifestyle. It is difficult to clearly define quality of diet due to the different given recommendations and standards for the consumption of individual nutrients. The current method to assess the correct balance of nutrients is to compare diet composition with the indicators of diet quality, which are created in relation to the diet pattern recognized as pro-health based on epidemiological studies. 

The Healthy Eating Index 2015 (HEI-2015) is one of the most up-to-date diet quality indicators and was developed on the basis of the 2015–2020 Dietary Guidelines for Americans (DGA) [1,2]. The HEI-2015 was recalculated to assess the quality of the diet of the study population which not only comprises the US population [3,4,5] but also the European [6,7], Asian [8] and South American populations [9]. Other demographics considered in the HEI-2015 included adults [10,11], children [12], the youth and young adults [13], and it also took into consideration people with specific health conditions such as pregnancy [14], cancer [15], obesity [16], diabetes [17] and depression [18]. However, there are only a few publications using HEI-2015 to assess diet quality in people with metabolic syndrome (MetS) [19,20].

The interest for studying MetS is growing among scientists due to a constantly increasing number of patients and doubts concerning the selection of various MetS diagnostic criteria. Over the last two decades, numerous authors and scientific organizations established the criteria for MetS diagnosis [21,22]. In the past, visceral obesity did not constitute a prerequisite for MetS diagnosis, however, is now included in diagnostic criteria for MetS. According to most scientific organizations, in order to confirm a diagnosis of MetS, at least three out of five criteria are required to be met by a given patient [23].

Major contributing factors for MetS are an unhealthy diet, which is characterized by a high consumption of saturated fatty acids (SFAs), trans fatty acid isomers, simple carbohydrates and salt, and a sedentary lifestyle [24]. In addition, excess calorie intake leads to oxidative stress and inflammation that may result in the development of cardiovascular disease (CVD) [25,26]. Understanding of diet quality of patients with MetS, from a holistic perspective, is limited. 

The aim of this study was to assess the diet quality, as measured by the HEI-2015, of MetS patients in comparison to healthy individuals. Specifically, we analysed which nutritional component contributed to the highest risk of MetS and we also searched the correlation between total HEI-2015 score and the individual MetS components. Lastly, we investigated the relationship between HEI-2015 scores and individual MetS components in the study and control groups.

## 2. Materials and Methods

### 2.1. Study Design and Patient Selection Criteria (Study Participants)

This was a cross-sectional study in which data was obtained from patients in the project implemented as part of the statutory activity (ST-854, Wroclaw Medical University) and was used to form a study group. At the beginning, the study group consisted of 222 patients, but 7 persons were excluded from the study (lack of data and daily food rations <700 kcal or >5000 kcal per day). Ultimately, the study group included 215 patients, 127 women and 88 men, from whom nutrition interviews were collected in years 2013–2017. Patients with MetS were hospitalized at the Clinical Department of Internal Diseases and the Clinical Department of Endocrinology in the 4th Military Hospital of Wroclaw.

MetS was defined according to American Heart Association, the National Heart, Lung, and Blood Institute and International Diabetes Federation (IDF) consisting of three or more of the following criteria: high-density lipoprotein cholesterol (HDL-c) < 50 mg/dL in women and <40 mg/dL in men or receiving drug treatment for HDL-c levels; triglycerides (TGs) ≥ 150 mg/dL or receiving drug treatment for elevated TG levels; fasting glucose ≥ 100 mg/dL or diagnosed with diabetes; waist circumference (WC) ≥ 80 cm in women for IDF and ≥94 cm in men for IDF; blood pressure ≥ 130/85 mmHg or diagnosed with hypertension [23,27].

The control group came from data obtained as part of the 6-year follow-up in Prospective Urban and Rural Epidemiology (PURE) Poland study, from whom nutritional interviews and other data were collected in 2013–2016. The control group consists of people who were not diagnosed with MetS, compared to the study group. The control group had fewer than three components of the MetS, taking into account the criteria of MetS as defined by AHA/NHLBI and IDF [23,27]. We noticed that 746 people were not diagnosed with MetS. Subsequently, 422 people with incomplete data were excluded from the study (lack of nutritional questionnaire, anthropometric measurements and body weight measurement). Ultimately, people who reported implausible daily food rations (<700 kcal or >5000 kcal per day) were excluded from the study. Finally, the control group consisted of 320 healthy people, 210 women and 110 men. 

The study was approved by the Bioethics Committee of the Wroclaw Medical University (no. KB–306/2018).

### 2.2. Clinical (Biochemical) and Anthropometric Evaluation

For the study group, blood chemistry tests for TGs, HDL-c and fasting blood glucose levels were obtained from the patient’s medical history during hospitalization. Moreover, antihypertensive and hypoglycaemic drugs, as well as other drugs affecting the lipid panel parameters, were considered based on the patient’s medical history.

In addition, the systolic blood pressure (SBP) and diastolic blood pressure (DBP) were measured using a certified digital sphygmomanometer in the studied group. The blood pressure measurement was performed twice in a sedentary position. The arm cuff was put on the upper right arm, which had no clothes on, at the level of the heart.

In order to evaluate the nutritional status of the examined patients, anthropometric measurements were performed, which included body weight, body height and waist circumference measurements. Body weight was measured with an accuracy of 0.1 kg using the Tanita HR-001. Body height was measured with an accuracy of 0.5 cm. Waist circumference was measured between the iliac crest and the last costal arch with the use of an anthropometric tape that was accurate to 0.5 cm; with waist circumference below 80 cm in women and below 94 cm in men considered normal [23,27]. 

In the control group, questionnaire, anthropometric parameters, laboratory tests (TGs, HDL-c, glucose levels), and blood pressure measurements, were recorded as part of the PURE study.

### 2.3. Dietary Assessment

A nutritional analysis using semi-quantitative food frequency questionnaire (FFQ), which comprised 154 questions, was used to evaluate the nutritional habits of both groups [28]. The questions involved the consumption of food, dishes and beverages, to which mean portions in household measures were assigned. Participants determined the frequency of consumption of individual products within the last year by selecting one of the following answers: never or less than once a month, 1–3 times a month, once a month, 2–4 times a week, 5–6 times a week, once a day, 2–3 times a day, 4–5 times a day, and 6 or more times a day. The mass of consumed products, expressed in household measures, were specified with the use of “Album of photographs of food products and dishes” [29]. Data concerning the mass of consumed portions of individual products and dishes were calculated per day and on the basis of the nutritional value of daily food rations of the patients using “Food Composition Tables” [30]. Mean food energy and content of selected nutrients (above 80 such as total protein, total fats, total carbohydrates, saturated fats, sodium, vitamin D and others) in daily food rations were calculated.

### 2.4. Diet Quality

Diet quality was assessed by calculating the HEI-2015. The HEI-2015 was developed by the US Department of Agriculture and the National Cancer Institute [1]. HEI-2015 was designed to evaluate concordance with the 2015–2020 DGA [2]. HEI-2015 comprises 13 dietary components, with total scores ranging from 0 (nonadherence) to 100 (optimal adherence) [31]. Components are divided into two groups—adequacy components that are recommended for inclusion in a healthy diet and moderation components that should be consumed sparingly [1]. There are 9 adequacy components: total fruits, whole fruits, total vegetables, greens and beans, whole grains, dairy, total protein foods, seafood and plant proteins, and fatty acids. The adequacy components score higher when more is consumed. There are also 4 moderation components: refined grains, sodium, added sugars, and saturated fats [31]. Less intake of these components results in a higher score. The components were calculated per 1000 kcal/d, with the exception of saturated fats and fatty acid. Fatty acids are calculated as ratio of unsaturated to saturated fatty acids [1,31]. The maximum score for individual components is 5 or 10 points. For groups of food products, except for refined grains—0 points will be awarded to a diet in which there are no products in the group. For the components: sodium, added sugars, saturated fatty acids and refined grains—0 points will be awarded when their daily food ration is higher than the threshold specified in the indicator [1,31].

### 2.5. Other Covariates 

Mean values of body mass index (BMI) were calculated. The BMI status was evaluated according to the World Health Organization (WHO) criteria. Patients were classified as underweight (BMI < 18.5 kg/m^2^), normal weight (BMI 18.5–24.9 kg/m^2^), overweight (BMI 25–29.9 kg/m^2^), or obese (BMI ≥ 30 kg/m^2^) [32]. 

### 2.6. Statistical Analysis

Descriptive data were presented as numbers and percentages for categorical variables and as the mean and standard deviation for numerical variables. Data normality was tested using Kolmogorov-Smirnov and Shapiro-Wilk’s tests. Chi-square test and Fisher’s exact tests were used for categorical data. The risk estimates, such as the odds ratio (OR) together with the 95% confidence intervals (CIs), were used to assess the risk of a given factor. The Mann-Whitney U test was used for subgroup analysis of non-normally distributed variables. In multiple group comparisons of numerical variables, the Kruskal-Wallis test was used for non-normally distributed variables. Spearman's rank-order correlation coefficients were calculated for diet quality scores. In addition, total HEI-2015 scores were divided into quintiles. The statistical significance level was set to *p* ≤ 0.05. Analysis were performed using Statistica v. 13.3 software (TIBCO Software Inc., Palo Alto, CA, USA, 2017).

Radar graphs were created to provide a visual representation of how the study group and control group in quintile 1 and quintile 5 achieved their HEI-2015 scores [33]. The same visual representation was constructed for men and women in whole populations (no division into groups). The radar graph simultaneously showed information about each component calculated for the HEI-2015 score [34]. A perfect HEI-2015 score was displayed as a yellow dotted line around the border of the radar graph. Radar graphs were constructed using Microsoft Office Excel 2007 (Microsoft Corp, Redmond, WA, USA).

## 3. Results

### 3.1. Study Population and Characteristics

Baseline characteristics of all study participants (*n* = 535) and the mean values of the MetS components are presented in Table 1.

All participants were of European descent and their mean age was 58.39 ± 11.71 years. Of all respondents, 37% were male. The study group had a significantly higher BMI score, compared to the control group (32.05 ± 6.25 vs. 26.04 ± 4.12). Mean values for four of the five MetS components were significantly different between the groups. MetS patients had significantly higher mean TGs (*p* < 0.001), fasting glucose (*p* < 0.001) and waist circumference (*p* < 0.001), but lower mean HDL-c values (*p* < 0.001). 

Based on an assessment of the odds ratio of MetS, out of all of its components, low HDL-c concentrations were found to increase the likelihood of MetS the most, in the entire study population (approximately 127-fold). It is worth mentioning that in men, of all MetS components, low HDL-c concentrations increased the chances of MetS 342-fold, while in women it increased the chances almost 83-fold. In addition, high TG levels and abnormal blood glucose levels also significantly increased the chances of MetS in the total population (approximately 46 and 31-fold). Results of the risk assessment of the individual components on the occurrence of MetS are shown in Table 2.

### 3.2. HEI-2015

Mean scores from each component of the HEI-2015, by group and gender, are presented in Table 3. The number of points obtained for components such as seafood and plant proteins (*p* < 0.0001), refined grains (*p* = 0.002) and sodium (*p* = 0.13) were significantly lower in MetS patients than in the control group. Total HEI-2015 scores were also significantly lower in MetS subjects than for those in the control group (65.04 ± 9.71 vs. 66.75 ± 8.88). In contrast, women showed significantly higher scores for six of the thirteen components of the HEI-2015, compared to men: total fruits (*p* < 0.0001), whole fruits (*p* = 0.032), total vegetables (*p* = 0.038), green and beans (*p* = 0.003), whole grains (*p* = 0.018), refined grains (*p* = 0.005). Total HEI-2015 scores were significantly higher in women than in men (66.83 ± 8.99 vs. 64.75 ± 9.57).

### 3.3. Quintiles—HEI-2015

Table 4 compares the mean values of the MetS components in the fifth quintile of the HEI-2015 total score for male and female from the study and control groups.

In the fifth quintile was the group with the best dietary quality as measured by the HEI-2015. In the fifth quintile, women with MetS had significantly lower mean HDL-c concentrations (*p* < 0.0001) but higher TGs (*p* = 0.0002), fasting glucose (*p* = 0.0352) and waist circumference (*p* < 0.0001) compared to women without MetS. Men with MetS in the fifth quintile also had significantly lower mean HDL-c levels (*p* < 0.0001) and higher TGs (*p* = 0.0003), fasting glucose (*p* = 0.001), and waist circumference (*p* < 0.0001), compared to men without MetS. 

Table 5 shows the correlation between total HEI-2015 score and the individual MetS components, and it also shows the correlation between MetS components in the total population and in the study and control groups. Across the population, there was a weak positive correlation between HDL-c concentrations and total HEI-2015 scores and a weak negative correlation between waist circumference values and total HEI-2015 scores. However, these correlations were not found in the MetS group or the control group. In analysis of the whole population, most correlations were observed between waist circumference and individual MetS components: HDL-c concentration (negative correlation), TG concentration, fasting glucose, DBP and SBP (positive correlation). A moderate negative correlation was also noted between TG values and HDL-c concentrations. Furthermore, a strong positive correlation was observed between SBP and DBP values. Meanwhile, weak correlations were observed between fasting glucose and SBP, HDL-c, and TG values.

### 3.4. Radar Graphs

**Figure 1:**
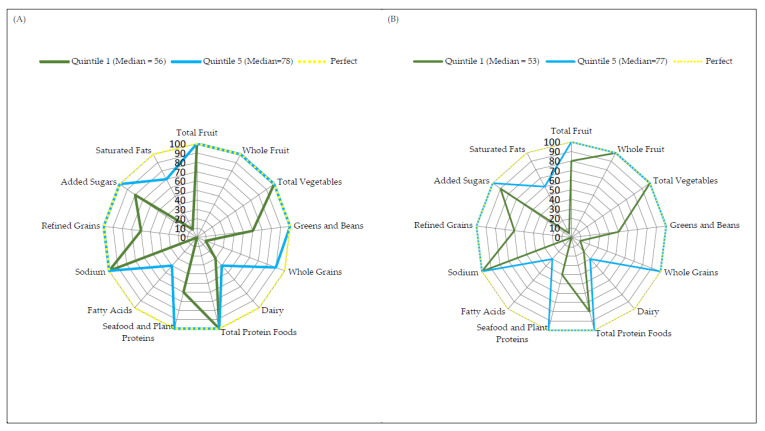
Radar graphs depicting a perfect score (100 points) and median component scores for (**A**) women and (**B**) men score in quintile one and quintile five for HEI-2015.

Radar plots, for men and women, show the median of each dietary component of the HEI-2015 scores within the first and fifth quintiles (Figure 1). The radar charts also show information on the score of each component from the HEI-2015 [34]. In the radar plots, the first quintile represents the group with the lowest diet quality, while the fifth quintile represents the group with the highest diet. The outer edge of the radar graphs, indicated by the yellow dashed line, represents the ideal HEI-2015 score, which is 100% of the maximum score for each component. The centre of the graph represents a score equal to 0% of the scores for any component.

Median HEI-2015 component scores for women in the first quintile were less than 50% for components such as whole grains, dairy, fatty acids and saturated fats; and they ranged from 60% to 80% for components such as greens and beans, seafood and plant proteins, refined grains and added sugars (Figure 1A). The median of the other components in the first quintile for women was 100% (total fruit, whole fruit, total vegetables, total protein foods, sodium). Meanwhile, the lowest median HEI-2015 scores in the fifth quintile for women were characterised by the components: dairy (40%), fatty acids (40%) and saturated fats (70%). Medians of the other components in the fifth quintile for women, apart from whole grains (90%), were 100% (Figure 1A). Median HEI-2015 component scores for men in the first quintile were less than 50% for components such as whole grains, dairy, seafood and plant proteins, fatty acids and saturated fats; and ranged from 50% to 80% for the components such as total fruit, greens and beans, total protein foods, and refined grains (Figure 1B). Median HEI-2015 component scores above 80% were recorded for whole fruit, total vegetables, sodium and added sugars. Meanwhile, the lowest median HEI-2015 scores in the fifth quintile for men were dairy (30%), fatty acids (30%) and saturated fats (60%) (Figure 1B). The medians of all other HEI-2015 components in the fifth quintile for men were 100% (Figure 1B). When comparing the median for HEI-2015 total scores in the female and male groups for the first and fifth quintile, women had a higher median HEI-2015 total score than men (Figure 1).

**Figure 2:**
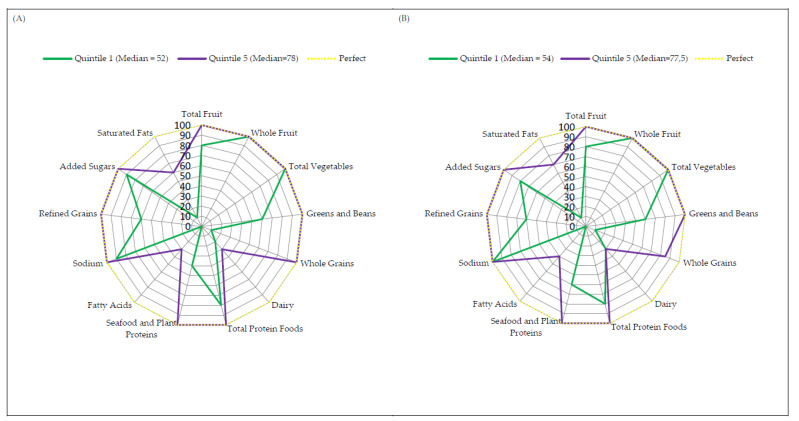
Radar graphs depicting a perfect score (100 points) and median component scores for (**A**) study group and (**B**) control group score in quintile one and quintile five for HEI-2015.

Analogous radar plots of the study and control group are shown in Figure 2. Median HEI-2015 component scores for the study group in the first quintile were 40% or less for components such as whole grains, dairy, seafood and plant proteins, fatty acids and saturated fats (Figure 2A). For the control group, the median component scores in the first quintile were 40% or less for: whole grains, dairy, fatty acids and saturated fats (Figure 2B). For the components: total fruit, greens and beans, total protein foods and refined grains, the median HEI-2015 component scores in the first quintile for the study group ranged from 60% to 80% (Figure 2A). For the control group in the first quintile, median scores of 60 to 80% were recorded for the total fruit, greens and beans, total protein foods, seafood and plant proteins, refined grains and added sugars components (Figure 2B). In the study group, a median of 90% or more was found for whole fruit, total vegetables, sodium and added sugars (Figure 2A). In contrast, in the control group, a median of 100% was recorded for whole fruit, total vegetables and sodium (Figure 2B). The lowest median HEI-2015 scores in the fifth quintile for the study and control group were for the dairy (30%; 30%), fatty acids (30%; 40%) and saturated fats components(60%; 70%) (Figure 2). The medians of the other HEI-2015 components in the fifth quintile for the study and control group (except whole grains in the control group) were 100% (Figure 2). Comparing the median for total HEI-2015 scores in the study and control groups for the first quintile, the group with MetS had a lower median total HEI-2015 score than the control group (52 vs. 54). However, MetS group in quintile five had a slightly higher median total HEI-2015 score than the control group (78 vs. 77.5).

## 4. Discussion

In our study, excessive body weight was found in 92.1% of patients with MetS and in 56.8% of those without MetS. Ma et al. [35], similar to our study, showed that patients with MetS had significantly higher mean BMI values than those in the control group (27.2 ± 3.6 vs. 23.9 ± 3.5). 

Several studies have shown most of the mean values of MetS components (waist circumference, SBP, DBP, fasting glucose and TGs) to be significantly higher in people with MetS compared to the control group, while mean values of HDL-c concentrations were significantly lower compared to the group without MetS [35,36,37]. Guembe et al. [36] concluded from their study that MetS was independently associated with CVD risk and with mortality associated with both cardiovascular and all-cause mortality.

A key finding of this study is that low HDL-c concentrations increased the risk of MetS the most in the total population. We also found that women and men with MetS with the highest diet quality (fifth quintile), as measured by the HEI-2015, had significantly lower mean HDL-c values than women and men without MetS (quintile five). Indeed, a positive correlation was found between HDL-c concentrations and total HEI-2015 scores in the overall population. Furthermore, a negative correlation was found between waist circumference and total HEI-2015 scores in the total population. However, these correlations were not found in the study group or the control group. A study by Liu et al. [38] found that the incidence of MetS increased in parallel with low HDL-c concentrations. Low HDL-c concentrations are a significant risk factor for MetS which increases the risk for atherosclerotic CVD [39]. HDL-c concentrations may be a key factor in the prevention of MetS. Therefore, appropriate therapeutic management to increase HDL-c levels may be of key importance in patients diagnosed with MetS.

According to guidelines from the European Society of Cardiology (ESC) and the European Atherosclerosis Society (EAS), lifestyle and dietary changes influenced lipoprotein levels which include total cholesterol, low-density lipoprotein cholesterol (LDL-c), TGs and HDL-c [40]. Evidence-based dietary interventions to increase HDL-c concentrations include avoiding trans isomers of unsaturated fatty acids in the diet, reducing dietary carbohydrate intake and replacing it with unsaturated fatty acids, moderate alcohol intake in patients for whom this may be a consideration (for patients with elevated TGs, no alcohol consumption is recommended), and reducing excess body [41,42,43,44,45,46]. These interventions resulted in a 5–10% increase in HDL-c concentration [40]. However, increasing physical activity had the most beneficial effect on increasing HDL-c concentrations (>10%) [47,48]. For example, 25–30 km of walking per week (or other aerobic physical activity) increased HDL-c concentrations by 3.1–6 mg/dL (0.08–0.15 mmol/L) [49]. According to the HEI-2015, diet quality of MetS patients had a lower score than controls subjects. Furthermore, in our study, and those of others, it was observed that the quality of women’s diets was better than the quality of men’s diets [5,20].

Consistent with our findings, Panizza et al. [5] found three components with the lowest median HEI-2015 scores were the same for individuals in the first and fifth quintile. The recurrent components with the lowest medians, both in our study and in that of Panizza et al. [5], were dairy and fatty acids. On the other hand, the third component with the lowest median in the study by Panizza et al. [5] was sodium, whereas we found this to be saturated fats.

The two component, whole fruit and total vegetables, with the highest median HEI-2015 scores (100%) were the same for individuals in the first and fifth quintile, for both men and women and in the study and control groups. The maximum HEI-2015 scores for whole fruit and total vegetables were ≥0.4 cup/1000 kcal and ≥1.1 cup/1000 kcal, respectively. All vegetables were included in the total vegetables component scores, including potatoes, which may have contributed to the higher scores obtained in the HEI-2015 indicator. Data from the National Health and Nutrition Examination Survey 2001–2018 showed that people who consumed potatoes in various forms had significantly higher total HEI-2015 and higher component scores (total vegetables, total protein foods, refined grains) than those who did not consume potatoes [50]. Therefore, we can conclude that the consumption of potatoes may have contributed to the high score for total vegetable intake in the current study.

The burden of CVD can be minimised by dietary modification, with a predominance of plant protein, a lower intake of SFA, and a reduction in animal protein from highly processed foods [51]. Researchers from the International Lipid Expert Panel presented a position statement explaining the differential effects of animal protein compared to plant protein on cardiometabolic risk factors [52]. Accordingly, animal protein from highly processed products and red meat increased the risk of CVD [52]. In contrast, animal protein sources such as poultry, fish and dairy products reduced the risk of CVD [52]. Furthermore, plant protein, particularly from sources such as soya, nuts and nutraceuticals also reduced the risk of CVD [52]. In the current study, individuals with MetS had significantly lower scores for seafood and plant proteins than the control group. Also, median HEI-2015 components scores in the first quintile were low for dairy (20%) and seafood and plant proteins (40%) in patients with MetS. Preservatives such as nitrates and the high sodium content of processed meat may have the strongest association with CVD risk [52,53]. The results of a study by Azemati et al. [54] suggested that including a significant amount of plant protein as part of total protein in the diet has a beneficial effect on cardiometabolic risk factors such as waist circumference and fasting glucose levels. The authors of numerous studies have also found that adherence to vegetarian dietary patterns is associated with a lower risk of developing MetS [55,56,57] and that plant protein may have a protective effect in the prevention of excessive weight and obesity in the general population [58].

The food groups among the HEI-2015 components that are sources of fibre, and particularly soluble fibre are: whole fruit, total vegetables, green and beans and whole grains. From these four components, patients with MetS in the first quintile had the lowest median for whole grains. Some studies have shown that increasing dietary fibre, especially the soluble fraction, improves the glycaemic response (lowers postprandial glucose) and had hypocholesterolaemia effects intake in MetS patients [59]. Indeed, consuming higher amounts of dietary fibre may reduce abdominal obesity by regulating energy homeostasis and altering gut microflora [60,61]. A meta-analysis by Chen et al. [62] found that those with the highest dietary fibre intake had a significantly reduced risk of MetS compared with those with the lowest dietary fibre intake. The pooled OR was 0.70 (95% CI: 0.61–082), but there was high heterogeneity (*p* < 0.001, I2 = 74.4%). Although the authors observed an inverse relationship between dietary fibre intake and the risk of MetS, they suggested the need for further prospective studies to further verify the relationship [62].

The lowest median HEI-2015 components scores were recorded for two of the four moderated components, fatty acids and saturated fats, in MetS patients with the lowest and the highest dietary quality (from the first and fifth quintiles). It is important in MetS diet therapy to limit the intake of SFAs due to their cholesterol-raising effects [63]. Excessive intake of SFAs led to the development of CVD in MetS due to the presence of increased oxidative stress and inflammation [52,64]. Numerous authors have shown that reducing SFAs and replacing them in the diet with unsaturated fatty acids, including monounsaturated fatty acids (MUFA) and/or polyunsaturated fatty acids (PUFA), was associated with a lower risk of cardiovascular incidents [65,66] and type 2 diabetes [67]. Also, replacing SFAs in the diet with carbohydrates from whole grain cereal products prevented CVD in MetS patients [66]. 

An adequate intake of n-3 PUFA from the diet is necessary, and a deficiency of these acids increased the risk of developing CVD [68]. N-3 PUFA played an important role in dietary therapy for MetS patients, as they exhibited antiarrhythmic, anticoagulant and anti-inflammatory effects. In addition, they lowered blood pressure, protected the endothelium, decreased LDL-c, reduced TG concentrations, and increased plasma HDL-c concentrations [67]. Of all the n-3 PUFAs, docosahexaenoic acid (DHA) and eicosapentaenoic acid (EPA) play the most important role in MetS dietary therapy, as they are the most responsible for proper cardiovascular function. However, DHA and EPA intake in MetS patients should be controlled due to their strong effects on coagulation and fibrinolysis [24]. According to ESC and EAS guidelines for the management of dyslipidaemia, the energy intake of n-3 PUFA should be less than 10% to minimise the risk of plasma lipid peroxidation and to avoid clinically significant reductions in HDL-c concentrations [69]. 

Four of the thirteen of HEI-2015 components, sodium, refined grains, added sugars and saturated fats, are most often found in ultra-processed foods (UPFs). Martínez Steele et al. [70] in Poisson regression models with robust variance adjusted for age, sex, race/ethnicity, family income, education, physical activity and smoking, to show significant linear association between the dietary contribution of UPFs and the prevalence of MetS. They also noticed that a diet with UPF intake > 71% was associated with a 28% higher incidence of MetS compared to a UPF intake of <40% [70]. There is increasing evidence that consuming highly processed foods is associated with MetS. In the relationship between UPFs and MetS, both the nutritional composition of UPFs (source of sodium, added sugars, saturated fatty acids, a small amount of fibre) and the presence of other substances added during production to UPFs are important [70]. The presence of additives and emulsifiers, among others, in UPF may adversely affect the gut microbiota and lead to inflammation [71]. Inflammation can lead to clinical progression of MetS in patients with excessive body weight [72]. In addition, UPFs are characterized by a lower satiety and a high glycaemic response [73], which is particularly disadvantageous for people with MetS who have been diagnosed with diabetes.

It is worth focusing on nutritional education and tailoring it to specific information regarding the food groups and nutrients in diets of MetS patients who have the lowest diet quality. The lowest median scores for the dairy, fatty acids, and saturated fats components of the HEI-2015 were the same for subjects in the first and fifth quintile, in both the study and control groups. In this case, it seems very important to educate all groups about the most poorly fulfilled components in order to improve their diet quality and prevent diet-related diseases.

Our study had several limitations. It was a retrospective observational study and the groups were not randomised. Also, the study included a population from only one country. A further limitation of the study was the use of the FFQ questionnaire to collect dietary data from the study population, since this could have led to bias in the results. However, it should be noted that the FFQ was validated for the Polish population studied. Data from the MetS patient group were only collected at the start of the study, and it was not possible to further assess the impact of the diet on, the implementation of nutritional education, patient deterioration or patient mortality. 

In future studies, it would be worth considering whether the diet of people with MetS changes significantly once MetS is established. There are no ideal dietary recommendations for patients with MetS due to the presence of different components in these individuals. Indeed, earlier recommendations often focused on the nutrients in the diet, whereas recommendations currently focus on a broader approach.

## 5. Conclusions

The HEI-2015 is a useful tool for the assessment of dietary quality of MetS patients, and opens up the opportunity to take a holistic approach to a patient’s diet without focusing on single dietary components. Using the HEI-2015, it is possible to identify dietary components that MetS patients have the most difficulty with in order for their diet to meet the objectives of a healthy, balanced diet.

Based on our study, the diet of MetS patients, as assessed by the HEI-2015, was found to be of lower quality than that of healthy individuals. Individuals with the lowest diet quality scores (from the first quintile) in both the study and control groups had the lowest scores for the same four components of the HEI-2015 index, which were whole grains, dairy, fatty acids and saturated fats. In MetS patients, reducing SFAs and increasing PUFAs and MUFAs, whole grain products and dairy products, may help to improve the diet of those with the lowest diet quality.

A major contributing component of MetS that influenced its occurrence was low HDL-c concentrations. Therefore, the key to preventing MetS appears to be achieving normal HDL concentrations.

## Figures and Tables

**Table 1:** Characteristic of participants of the study and mean metabolic syndrome components for study and control group.

	Total ** (*n* = 535)	Study Group (*n* = 215)	Control Group (*n* = 320)	*p* Value
Characteristic	Mean score ± SD			
Age [lata]	58.39 ± 11.71	58.48 ± 14.65	58.33 ± 9.26	0.509
BMI [kg/m^2^]	28.45 ± 5.87	32.05 ± 6.25	26.04 ± 4.12	<0.001
	*n* (%)			
Male gender	198 (37.0%)	88 (40.9 %)	110 (34.4%)	0.124
Metabolic syndrome components	Mean score ± SD			
HDL-c (mg/dL)	57.06 ± 20.93	42.35 ± 15.23	66.95 ± 18.27	<0.001
TGs (mg/dL)	122.21 ± 71.39	163.45 ± 89.98	94.50 ± 34.41	<0.001
glucose (mg/dL)	101.16 ± 32.78	116.95 ± 46.47	90.55 ± 8.32	<0.001
waist circumference (cm)	92.56 ± 16.51	104.94 ± 15.11	84.25 ± 11.43	<0.001
systolic blood pressure (mm Hg)	133.68 ± 17.08	133.68 ± 18.27	133.68 ± 16.27	0.833
diastolic blood pressure (mm Hg)	83.31 ± 10.29	83.87 ± 11.20	82.93 ± 9.64	0.48

** Total—both the study group and control group; BMI—body mass index; HDL-c—high-density lipoprotein; TGs—triglycerides.

**Table 2:** Odds ratios with 95% confidence interval for the association between the risk of metabolic syndrome and its components.

Variables	Total **(*n* = 535)	Women (*n* = 337)	Men (*n* = 198)
	Odds ratio (95% CI)	Odds ratio (95% CI)	Odds ratio (95% CI)
Low HDL-c *	126.77 (63.31–253.82)	82.72 (37.48–182.55)	342.00 (74.39–1572.23)
High TG *	45.84 (25.61–82.06)	43.63 (20.36–93.50)	50.03 (20.07–124.73)
Hyperglycaemia *	30.58 (18.16–51.49)	29.60 (15.13–57.90)	31.71 (13.81–72.82)
Increased WC	16.20 (9.30–28.22)	16.05 (7.47–34.51)	18.95 (8.29–43.29)
High BP and/or SBP *	5.50 (3.22–9.39)	5.40 (2.85–10.21)	5.40 (1.98–14.70)

* or drug treatment; ** Total—both the study group and the control group. HDL-c: high-density lipoprotein cholesterol (<40 mg/dl in males; <50 mg/dl in females); TGs: triglycerides (≥150 mg/dl); hyperglycaemia (≥100 mg/dl); WC: waist circumference (≥94 cm for men and ≥ 80 cm for women); BP: blood pressure (systolic ≥ 130 mm Hg and/or diastolic ≥ 85 mm Hg).

**Table 3:** HEI-2015 components scores for study group and control group (mean and standard deviation).

	Study Group (*n* = 215)		Control Group (*n* = 320)		*p* Value	Women (*n* = 337)		Men (*n* = 198)		*p* Value
HEI-2015 (0–100)	Median	Range	Median	Range		Median	Range	Median	Range	
	66.0	41.0–93.0	68.0	41.0–89.0		68.0	41.0–93.0	65.0	41.0–89.0	
HEI-2015 Component scores (maximum score)	Mean score ± SD					Mean score ± SD				
Total fruits (5)	4.49 ± 0.94		4.62 ± 0.85		0.074	4.68 ± 0.79		4.38 ± 1.01		<0.0001
Whole fruits (5)	4.88 ± 0.50		4.91 ± 0.46		0.558	4.93 ± 0.41		4.84 ± 0.57		0.032
Total vegetables (5)	4.83 ± 0.48		4.75 ± 0.62		0.117	4.82 ± 0.51		4.72 ± 0.65		0.038
Green and beans (5)	3.91 ± 1.32		3.97 ± 1.24		0.790	4.07 ± 1.24		3.74 ± 1.31		0.003
Whole grains (10)	4.67 ± 3.89		5.07 ± 3.61		0.104	5.20 ± 3.68		4.42 ± 3.77		0.018
Dairy (10)	3.14 ± 1.32		3.12 ± 1.38		0.852	3.21 ± 1.38		2.99 ± 1.30		0.063
Total Protein Foods (5)	4.53 ± 0.70		4.38 ± 0.90		0.128	4.41 ± 0.87		4.49 ± 0.76		0.386
Seafood and plant proteins (5)	3.38 ± 1.48		3.98 ± 1.28		<0.0001	3.71 ± 1.43		3.79 ± 1.34		0.641
Fatty acids (10)	1.76 ± 2.20		1.67 ± 2.12		0.721	1.70 ± 2.16		1.72 ± 2.14		0.732
Refined grains (10)	8.33 ± 2.33		8.68 ± 2.43		0.002	8.69 ± 2.38		8.27 ± 2.40		0.005
Sodium (10)	8.56 ± 2.30		9.08 ± 1.66		0.013	8.85 ± 1.96		8.90 ± 1.96		0.892
Added sugars (10)	8.81 ± 1.79		8.78 ± 1.57		0.208	8.84 ± 1.58		8.71 ± 1.78		0.669
Saturated fats (10)	3.73 ± 2.95		3.75 ± 3.06		0.909	3.73 ± 3.08		3.77 ± 2.91		0.845
Total score (100)	65.04 ± 9.71		66.75 ± 8.88		0.015	66.83 ± 8.99		64.75 ± 9.57		0.006

**Table 4:** Mean metabolic syndrome components for women and men with and without MetS in the fifth quintile of HEI-2015 total score.

	Women with MetS (*n* = 20)	Women without MetS (*n* = 43)	
	Quintile 5	Quintile 5	*p* Value *
Median HEI-2015 score	80.0	77.0	0.030
Metabolic syndrome components	Mean score ± SD		
HDL-c (mg/dL)	46.95 ± 20.29	75.65 ± 15.66	<0.0001
TGs (mg/dL)	152.75 ± 73.89	88.98 ± 31.58	0.0002
glucose (mg/dL)	108.20 ± 29.85	88.33 ± 7.07	0.0352
waist circumference (cm)	102.15 ± 17.24	79.33 ± 11.13	<0.0001
systolic blood pressure (mm Hg)	138.40 ± 15.53	133.31 ± 17.50	0.223
diastolic blood pressure (mm Hg)	84.45 ± 11.82	82.65 ± 10.55	0.451
	Men with MetS (*n* = 14)	Men without MetS (*n* = 23)	
	Quintile 5	Quintile 5	*p* Value *
Median HEI-2015 score	77.5	77.0	0.270
Metabolic syndrome components	Mean score ± SD		
HDL-c (mg/dL)	35.86 ± 7.13	58.61 ± 15.16	<0.0001
TGs (mg/dL)	201.07 ± 130.49	100.09 ± 39.70	0.0003
glucose (mg/dL)	133.07 ± 47.24	95.42 ± 11.30	0.001
waist circumference (cm)	107.86 ± 10.20	90.91 ± 9.30	<0.0001
systolic blood pressure (mm Hg)	140.64 ± 17.84	137.26 ± 17.21	0.377
diastolic blood pressure (mm Hg)	85.68 ± 13.06	84.63 ± 9.54	0.588

* U Mann-Whitney’s test.

**Table 5:** Spearman's correlations between HEI-2015 total score and metabolic syndrome components.

		HEI-2015 Total Score	Glucose (mg/dL)	SBP (mm Hg)	DBP (mm Hg)	HDL-c (mg/dL)	TGs (mg/dL)	WC (cm)
Total **	HEI-2015 total score	-	−0.035	0.023	−0.029	0.120 *	−0.053	−0.103 *
	glucose (mg/dL)	−0.035	-	0.166 *	0.073	−0.262 *	0.229 *	0.315 *
	SBP (mm Hg)	0.023	0.166 *	-	0.643 *	−0.011	0.014	0.172 *
	DBP (mm Hg)	−0.029	0.073	0.643 *	-	−0.109 *	0.127 *	0.217 *
	HDL-c (mg/dL)	0.120 *	−0.262 *	−0.011	−0.109 *	-	−0.578 *	−0.596 *
	TGs (mg/dL)	−0.053	0.229 *	0.014	0.127 *	−0.578 *	-	0.456 *
	WC (cm)	−0.103 *	0.315 *	0.172 *	0.217 *	−0.596 *	0.456 *	-
Study group (with MetS)	HEI−2015 total score	-	0.034	0.092	0.011	−0.022	0.010	−0.024
	glucose (mg/dL)	0.034	-	0.157 *	0.053	−0.192 *	0.228 *	0.254 *
	SBP (mm Hg)	0.092	0.157 *	-	0.604 *	0.011	−0.052	0.157 *
	DBP (mm Hg)	0.011	0.053	0.604 *	-	−0.141 *	0.148 *	0.239 *
	HDL-c (mg/dL)	−0.022	−0.192 *	0.011	−0.141 *	-	−0.429 *	−0.251 *
	TGs (mg/dL)	0.010	0.228 *	−0.052	0.148 *	−0.429 *	-	0.176 *
	WC (cm)	−0.024	0.254 *	0.157 *	0.239 *	−0.251 *	0.176 *	-
Control group (without MetS)	HEI−2015 total score	-	−0.072	−0.029	−0.054	0.105	−0.014	−0.082
	glucose (mg/dL)	−0.072	-	0.171 *	0.079	−0.122 *	0.032	0.157 *
	SBP (mm Hg)	−0.029	0.171 *	-	0.672 *	−0.062	0.083	0.277 *
	DBP (mm Hg)	−0.054	0.079	0.672 *	-	−0.131 *	0.124 *	0.277 *
	HDL-c (mg/dL)	0.105	−0.122 *	−0.062	−0.131 *	-	−0.382 *	−0.395 *
	TGs (mg/dL)	−0.014	0.032	0.083	0.124 *	−0.382 *	-	0.282 *
	WC (cm)	−0.082	0.157 *	0.277 *	0.277 *	−0.395 *	0.282 *	-

* Significant r values (*p* < 0.05); ** Total—both the study group and control group.

## Data Availability

Not applicable.
